# A study of the histological behavior of a rabbit vocal fold after a hyaluronic acid injection

**DOI:** 10.1016/S1808-8694(15)31063-6

**Published:** 2015-10-22

**Authors:** Paulo Sérgio Lins Perazzo, André de Campos Duprat, Carmem Lancelotti, Fernanda Donati

**Affiliations:** 1 Master's degree student at the Sao Paulo Santa Casa Medical Science School, Director of the Clinic Otorrinos.; 2 Professor, Master and Doctor of the Otorhinolaryngology Department of the Sao Paulo Santa Casa Medical Science School.; 3 Director of the Pathology Department of the Sao Paulo Santa Casa Medical Science School.; 4 Third year resident of the Otorhinolaryngology Department of the Sao Paulo Santa Casa Medical Science School.; 5 This study was undertaken in the ENT and Morphology Departments of the Sao Paulo Santa Casa Medical Science School.

**Keywords:** inflammatory, vocal fold insufficiency, ocal cord, hoarseness, hyaluronic acid

## Summary

**T**he vocal fold structure is composed of tissues with many cells surrounded by the extra-cellular matrix. One of the most important components of the extra-cellular matrix is Hyaluronic Acid (HA). The aim of the study was to evaluate the inflammatory response of rabbit vocal folds after a local injection of Restylane® HA. **Methods**-Twelve adult male rabbits randomly received a 0.1 ml injection of Restylane® HA in one vocal fold and 0.1 ml of saline in the other vocal fold. The animals were prospectively subdivided into two groups; animals in one group were sacrificed after one week of follow-up and animals in the other group were sacrificed after 3 months. Slides were Hematoxylin-Eosin (HE), Masson Trichromic and Toluidine Blue stained. **Results-** Hyaluronic Acid was found microscopically in all specimens in both groups. There was more connective tissue surrounding HA, always associated with a mild inflammatory response. The longer exposure time did not increase the intensity of inflammation. Tissue necrosis and foreign body inflammatory reaction were not observed in both groups. **Conclusion-**The study suggests that HA is a good alternative as a filling material in vocal folds when treating glottal insufficiency.

## INTRODUCTION

Scientists studying the human voice have always sought a feasible alternative to correct glottal insufficiency. This entity is characterized by incomplete glottal closure -seen under stroboscopy - while emitting a sustained vowel at a comfortable intensity and pitch. Normal phonation depends, among other factors, on complete glottal closure and malleability of the vocal fold (VF) mucosa, which are the main determinants of good voice quality.

Professor Isshiki conducted experiments on excised larynxes and elegantly demonstrated the need for perfect glottal closure; in other words, an investigation of the initial glottic area with no subglottic air flow (Ag0). If this condition is not met, glottal closure will be imperfect, resulting in slits that cause the turbulent flow of infraglottic air instead of a physiological laminar air flow.^1^

The structure of laryngeal tissues, which is composed of layers with different viscoelastic properties, is essential for adequate voice production. Soft tissues that form the vocal folds (VFs) are far more complex than originally thought. They are formed by thin lubricated layers in intimate contact with the ipsilateral tissues, allowing the onset of VF wavelike movement at the moment of complete glottal closure. This phenomenon was well studied in past decades by Professor Hirano, who revolutionized our understanding of voice physiology with the body/cover theory.^2^

Various authors in recent papers have underlined the importance of the VF ultra-structure, emphasizing the extracellular matrix (EM), and have defined its protein components. These substances, which include hyaluronic acid (HA), are responsible for the viscoelastic properties of VFs.^3^, ^4^, ^5^

The EM is composed of fibrous proteins and interstitial elements. Fibrous proteins, which include elastin and collagen, provide support to the EM and give VFs the ability to withstand external pressure. Interstitial components include proteoglycans and glycoproteins, which envelop the fibrous elements. These components have biological features that influence cell function, such as osmosis, cell migration, cell differentiation, molecule transport, and molecule concentration. Based on these characteristics, we believe that manipulation of EM elements might result in the discovery of valuable treatments in a wide variety of VF conditions.

Minor quantitative changes in these macromolecules can result in a marked negative impact on the biomechanical properties of the VF extracellular matrix, such as oscillation and propagation of the mucous wave (mucoondulatory component).

An ideal substance to substitute live tissues has been reason for much medical research. For VF surgery this prospective ideal substance should have the following characteristics:

1 – be made of a single substance;
2 – be non-immunogenic;
3 – be non-toxic;
4 – not produce inflammation;
5 – able to be used as an injection with no major trauma;
6 – to remain in the host for a long period;
7 – to be biocompatible at the application site. As VFs are the host site in our case, this proposed substance should not interfere with VF oscillation.^6^, ^7^, ^8^

Various substances have been used in attempts to recompose glottal closure and the VF mucous wave. Brünings (1911) apud,^9^ described the first substance to be implanted in VFs, namely liquid paraffin for the treatment of patients with recurrent nerve palsy. Although the author described few complications, widespread use of paraffin resulted in significant side effects such as granulomas and migration of the injected material to lymphnodes of the neck.

Other synthetic substances have been used with similar aims, such as tetrafluoroethylene (Teflon) and silicone, all with undesirable side effects such as granulomas - which are hard to remove - and migration to other sites.^10^,^11^

These negative results have fostered the search for biologically compatible materials to be used as VF space fillers, among which are collagen and fat. Unfortunately, these materials have also shown undesirable effects such as resorption. Immunogenic effects have also been observed against bovine collagen. Fat required multiple applications due to intense resorption.^12^,^13^

Unpredictability in the resorption rate of these biological materials has encouraged a search for biocompatible materials with lower or absent resorption in host tissues. These studies included biological materials that have lower resorption rates after undergoing structural change; HA is one of these materials.

Various factors should be observed when choosing a biomaterial to be implanted in VFs. These include: ease of implantation, the type of immune response, migration of the material to undesired regions, the possibility of injecting a larger amount of material without causing harm to the host, and duration of the implant.^6^

Chan & Titze (1999) compared various VF implantable materials and showed that the viscoelastic properties of HA were similar to those of the normal VF lamina propria in men and women.

VF viscoelastic properties require a higher or lower phonation pressure to generate Bernoulli's phenomenon. It is assumed that lack of HA causes voice changes, as HA is the main component of the VF extracellular matrix responsible for its viscoelasticity. In this case replacing HA as a biological implant might be a solution for this group of conditions.

HA in its natural state has been used since the 1980s to improve healing of tympanic membrane perforations. Sten & Laurent (1987) used HA experimentally in rats with traumatic tympanic membrane perforation. This group was compared to another group of untreated rats, and the result was faster closure in the HA-treated group. This group also has less residual scarring.

Artificial forms of HA are widely used in other parts of the body, such as in joints, in the eye and intradermally to fill in facial lines and wrinkles. Various papers have shown that HA is not completely inert in biological tissues. There have been complications such as erythema immediately after HA application. Late reactions - between six and eight weeks - have also been noted, including significant inflammation, including subcutaneous abscesses. Lowe et al. (2001) declared, based on observation, that these agents clearly have the potential to cause skin reactions.

Duranti et al. (1998) found a 12.5% rate of local and transitory immediate adverse effects after intradermal injection of HA, the most significant being local swelling in the application site. When HA was used to alleviate joint pain, adverse effects were related to the original disease, not to the treatment.^18^

There have been few studies on HA used in VFs. Most papers about HA describe its use in plastic surgery, orthopedics and ophthalmology rather than otorhinolaryngology, which undoubtedly underlines the need for animals studies prior to application of this material in humans.

A first study on the use of HA in VFs was done in 1998 by Hallen et al., who investigated a mixture of a hyaluronic acid solution in its natural state with dextranomere microspheres (DiHA) injected in rabbit VFs. This material contains equal parts of microspheres (80 to 120μm dextranomeres) and a 1% HA solution. The dextranomere particles are dry and porous, consisting of a strongly hydrophilic three-dimensional structure linked to a dextran molecule. This study showed that application of DiHA produced collagen fiber neoformation enveloping dextranomere microspheres and a weak inflammatory reaction, but no foreign body reaction. The authors concluded that HA is a perfect vehicle for DiHA due to its viscoelasticity, allowing injection with no significant trauma.

The combination of HA with dextranomere microspheres resulted in longer stay of the material implanted in VF soft tissues, collagen formation, increased dextranomere microsphere permanence in tissues up to six months - the follow-up period after VF injection - and complete absorption of HA; finally, there was only a weak inflammatory reaction throughout the 6-month period.

Endogenous HA solutions do not have the necessary rheological properties (viscosity, elasticity, and stability) to be used in VF space-filling surgery; these solutions are not sufficiently viscoelastic and remain in soft tissues for short periods, which make them unsuitable for such procedures. A chemical process based on the reaction of divinyl sulphone (DVS) linked to the HA hydroxyl radical in an aqueous alkaline solution at room temperature to form a HA gel was used to improve the physical and chemical characteristics and the stability of HA. This process is named cross-linking of HA, which has given rise to trade names (Restylane® Hyalgan® and Hylaform®).^8^

Hallen et al. (1999) used rabbits to study the histological behavior of HA that had been extracted from the cockscomb and cross-linked (Hylan b gel), different from microsphere-containing DiHA. HA in its Hylan b gel form was injected in rabbit VFs. No foreign body reaction was seen, and the authors concluded that chemical modification of HA into the Hylan b gel form increased stay of this substance in VF tissues without causing significant inflammation or other adverse reactions. Based on these results, the authors concluded that this material might be useful to correct glottal insufficiency of any cause in humans.

Hertegard et al. (2002) compared HA Hylan b gel (cross-linked) and bovine collagen in a series of patients, concluding that Hylan b gel and bovine collagen may be safely used as injections for treating glottal insufficiency. Both methods showed improvement of voice quality parameters. Hylan b gel-treated patients showed the best results in maximum phonation time 12 months after treatment, compared with collagen-treated patients.

Scarring of the lamina propria causes serious phonation disorders by altering the VF viscoelastic properties and modifying phonation physiology. Constant research, therefore, has been conducted to find a substance that might fill in Reinke's space without altering its rheological properties.

We decided to study the histological behavior of another presentation of HA - extracted from a Streptococus culture (Restylane®) - based on the important influence HA has over the quality of viscoelastic properties of the VF lamina propria surface layer and the encouraging results from implanting Hylan b gel (extracted from the cockscomb) in rabbit and human VFs.

Careful control must be obtained when applying saline in the ipsilateral VF in rabbits, as this traumatic procedure in itself might cause local inflammation and give rise to fibroblast-mediated regeneration. This type of control was not made in previous studies with Hylan b gel (cockscomb). This paper aimed to assess the safety of using HA Restylane after injection in rabbit VF, to study its histological behavior, and to compare this behavior with the ipsilateral fold that received an injection of saline (control group). The Research Ethics Committee approved the design and execution of this study in protocol number 41, dated 01 July 2003.

## MATERIAL AND METHODS

### The product: Restylane®

Restylane® is currently sold in Europe and was approved under the rules of the Council Directive 43/42 /EEC ANNEX II. It is produced from an equine Streptococcus culture by fermentation with sugar; the fermented material is precipitated in alcohol, filtered and dried. HA chains are chemically stabilized by a cross-linking process and its molecules are altered by only 1%. The result is a 2% viscoelastic, transparent, injectable, non-water soluble gel at a 20g/ml concentration, with high affinity for preserved water, and the ability to gain volume and form hydrated polymers.

### Selection of animals

We selected twelve healthy New Zealand male adult rabbits weighing between 2.8 kg and 3.9 kg, obtained from the biotherium of the Sao Paulo Santa Casa Medical Science School (FCMSCSP).

The protocol followed the guidelines for experimental surgery of the FCMSCSP Research Ethics Committee.

### Surgical technique

Surgery was done under intramuscular anesthesia with Vetarnacolâ (ketamine chloridrate – 20 to 25mg/kg), Anasedanâ (xylazine chloridrate – 1.5 ml for each 10kg weight). No preanesthetic medication was used.

The animals were placed in decubitus on the surgical table with the front limbs parallel to the body. The larynx was presented by restraining the tongue with gauze to expose the hypopharynx; the larynx was then visualized using a Storz® zero degree nasal endoscope and a 200-Watt Storz® light source.

Under spontaneous breathing, we first injected 0.1ml of saline in one of the VFs chosen randomly, which was considered the control VF, followed by injection of 0.1ml of HA (Restylane®) in the ipsilateral VF. The fine needle we used was the 27 gauge 35 cm needle that is employed in spinal anesthesia.

Restylane® may be kept at room temperature; when opened, it must be used within hours. It may not be resterilized or used with other substances. It is easy to inject and there is no need for manual pressure or special syringes.

### Study groups

The study sample included 12 rabbits that were divided into 2 groups of 6 rabbits selected randomly. Definition of the groups was done after surgery by a draw based on an increasing number scale. The first group was sacrificed one week after the procedure (group I) and the second group was sacrificed after three months (group II).

The choice of which VF would receive the HA injection was defined at the beginning of surgery. The control was the VF receiving a saline injection.

### Histological study

Histology was done at the FCMSCSP pathology department. HA-injected or saline-injected (control) hemilarynxes were assessed by an examiner who did not know to which groups each belonged. Fixation was done with formaldehyde at 10% for 24 hours. Coronal three-micrometer thick sections through the VF membranous portion were made. Sections were hematoxilin-eosin (HE), Masson's trichrome, and toluidine blue stained.

Histology was done using a Zeiss® conventional optic microscope to assess inflammatory cell intensity and predominance, neovascularization, fibrogenesis and areas of necrosis.

The inflammatory infiltrate was assessed according to intensity using a weighting or ponderal index divided into four grades: Grade 0, absent; Grade 1, mild; Grade 2, moderate; and Grade 3, intense (Chart 1). Predominant cell types are also described: neutrophils (N), mast cells (M), lymphocytes (L), plasmocytes (P), histiocytes (H), eosinophils (E), lymphohistiocytic (LH), and lymphoplasmacytic (LP).

**Chart 1:** Intensity of the inflammatory infiltrate according to the presence of cells and intensity of fibrosis.

Grade 0	Absent
Grade 1	Mild
Grade 2	Moderate
Grade 3	Intense

Fibrogenesis was quantified using an intensity scale as follows: Grade 0, absent; Grade 1, mild; Grade 2, moderate; and Grade 3, intense (Chart 1). The fibrous pseudocapsule around the injected material was not considered in measurements of intensity of fibrosis. Neovascularization and areas of necrosis were described as absent (Grade 0) or present (Grade 1) (Chart 2).

**Chart 2:** Neovascularization and Necrosis.

Grade 0	Absen
Grade 1	Present

### Analysis of results

The intensity of VF inflammation after HA injection was compared to that in the ipsilateral VF for the two time periods: one week and three months after the procedure.

A comparative study of cell types (neutrophils, mast cells, lymphocytes, plasmocytes, histiocytes and eosinophils) found in HA-injected VFs compared to ipsilateral saline-injected VFs was done for the two time periods.

Fibrosis in HA-injected VFs was compared to that in ipsilateral saline-injected VFs for the two time periods.

The progression of analysis parameters (inflammation intensity and cellularity, neovascularization, necrosis, and fibrosis) was studied for both groups in the two time periods to detect changes in time. These may be seen in the following tables that show the groups and analysis parameters.

Two rabbits died before the seventh day. Rabbit number 01 presented acute respiratory failure immediately after surgery and died 24 hours later. Rabbit number 12 died on the third day of unknown causes.

**Group I:** One week

Identification of the animal (Order). Intensity of inflammation (Infiltrate): mild (1), moderate (2), intense (3). Predominant cells: lymphoplasmacytic (LP), lymphohistiocytic (LH), lymphocytic (L), neutrophilic (N). Neovascularization. Absent (0), Present (1). Fibrogenesis: absent (0), mild (1), moderate (2), intense (3). Necrosis: absent (0), present (1).

**Table 1:** Group I - vocal fold control.

Order	Infiltrate	Cell predominance	Neovascularization	Fibrogenesis	Necrosis
7	1	N	0	0	0
8	0	-	0	0	0
9	0	-	0	0	0
10	0	-	0	0	0
11	0	-	0	1	0

**Tablel 2:** Group I - vocal fold with hyaluronic acid.

Order	Infiltrate	Cell predominance	Neovascularization	Fibrogenesis	Necrosis
7	2	LH	1	3	0
8	1	LP	1	1	0
9	1	L	1	1	0
10	2	LH	0	0	0
11	1	LH	1	1	0

Identification of the animal (Order). Intensity of inflammation (Infiltrate): mild (1), moderate (2), intense (3). Predominant cells: lymphoplasmacytic (LP), lymphohistiocytic (LH), lymphocytic (L), neutrophilic (N). Neovascularization. Absent (0), Present (1). Fibrogenesis: absent (0), mild (1), moderate (2), intense (3). Necrosis: absent (0), present (1).

Identification of the animal (Order). Intensity of inflammation (Infiltrate): mild (1), moderate (2), intense (3).

**Group II** – 3 months

**Table 3:** Group II - vocal fold control.

Order	Infiltrate	Cell predominance	Neovascularization	Fibrogenesis	Necrosis
2	0	-	0	0	0
3	0	-	0	0	0
4	0	-	0	0	0
5	0	-	0	0	0
6	0	-	0	0	0

**Table 4:** Group II - vocal fold with hyaluronic acid

Order	Infiltrate	Cell predominance	Neovascularization	Fibrogenesis	Necrosis
2	1	L	0	1	0
3	0	-	0	1	0
4	1	LH	0	1	0
5	1	LH	1	1	0
6	0	-	0	1	0

## DISCUSSION

HA Restylane® was chosen due to ease in handling, ready availability in the Brazilian market, storage at room temperature, and for being the first FDA-approved HA (December 2003). It is an affordable product that is available as a cross-linked gel and is a natural material with significant biocompatibility and low immune reactivity; it is also non-carcinogenic. The product does not cause significant infection, and is an excellent choice in surgery for correcting free edge of VF scars or as a space filler.^19^, ^20^, ^21^, ^22^

Macroscopically it was difficult to define which of the VF had been HA-injected. Thus, in four paraffin-included specimens, deeper sections had to be made to find HA.

Curiously in rabbit 10 we found that HA was located in two different sites, the submucosa and muscle. Probably during application, the needle moved at the moment HA was being injected. It is almost impossible to obtain complete VF immobility only with sedation; under spontaneous breathing it is difficult to inject HA in VFs with precision and reproducibility, as VFs move very rapidly in certain sites.^23^ In our study the substance was found in different levels of the VFs.

Rabbit 5 presented acute respiratory failure and received maximum concentration O_2_ ventilatory support, eventually progressing with no further problems. These animals are very sensitive to handling of the glottis, easily developing glottal edema which may progress to acute respiratory failure and eventual death of the animal.

Rabbits number 01 and 12 died of acute infection, which was seen on histopathology. Sepsis was clearly demonstrated by the presence of many piocytes and significant amounts of necrotic material. During the final moments of this process, these rabbits were tachypneic and probably developed severe glottal edema that caused their deaths. Although the procedure is a simple injection in VFs, careful antisepsis of materials used in the procedure is required, as in any other experimental study.^23^,^24^

**Figure 1:**
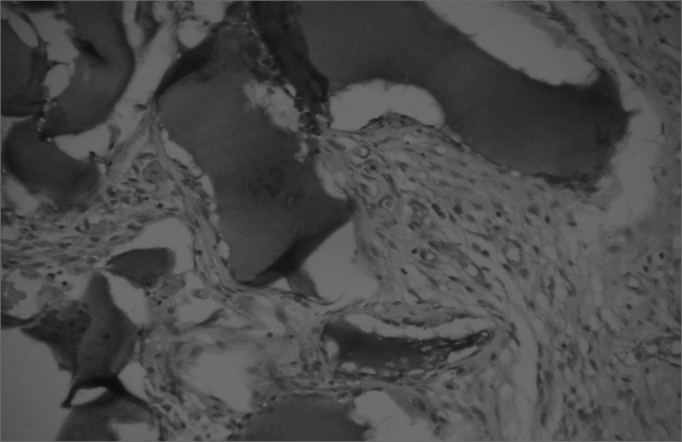
Rabbit 7 - left vocal fold, highlighting hyaluronic acid framented by fibrosis and vascular neoformation. (x 100, HE).

**Figure 2:**
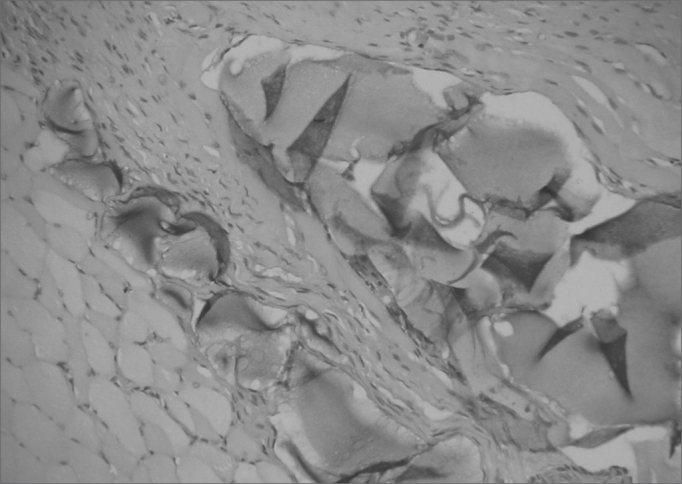
Rabbit 10 - right vocal fold, presence of blocks of hyaluronic acid within the muscle, enveloped by a thin pseudocapsule (x 100, HE).

**Figure 3:**
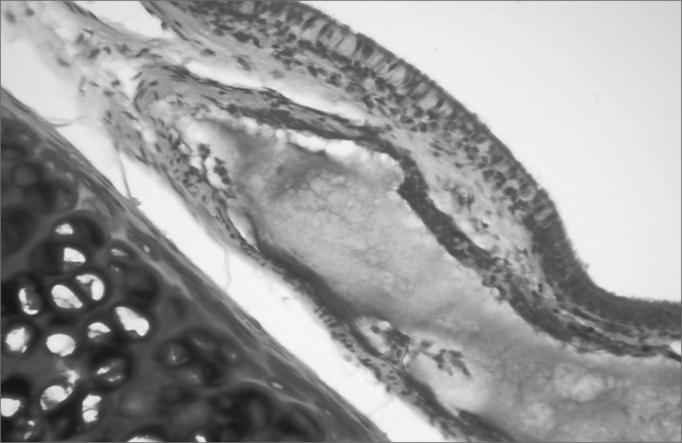
Rabbit 3 - right vocal fold, note hyaluronic acid in the lamina propria, with mild fibrosis. The highlight shows the presence of cartilage. (x 100, HE).

The aim of injecting a substance in VFs in cases of VF palsy is to increase the volume of palsied VFs and scars and to fill in Reinke's space, preserving its viscoelastic and rheological properties. HA Restylane® is a promising substance in this context; it did not migrate or degrade, and is water insoluble. In this study we found that there was perfect integration between HA and host tissues (Figures 1 and 2), with collagen regeneration of connective tissue, mild fibrosis, and VF augmentation due to formation of new connective tissue (Figure 3).

A few empty spaces were observed within HA blocks in some sections in both groups. This probably is due to 'suction' of tissues enveloping the gel during the histological fixation process, since the gel is water-insoluble; this finding has been previously reported in literature.^23^

**Figure 4:**
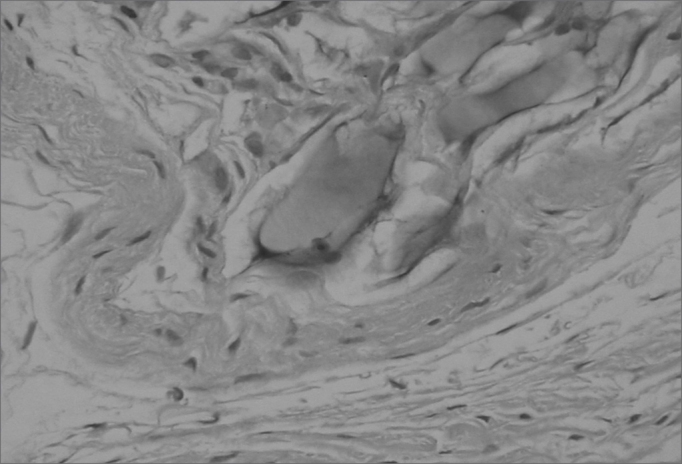
Rabbit 5 - left vocal fold, predominance of a thin pseudocapsule enveloping hyaluronic acid within the thyroarytenoid muscle. There is a mild macrophage infiltrate. (x 100, HE).

Different from collagen, HA Restylane® was well tolerated and remained stable throughout the study period in all tissues (Figure 3), presenting the same histological pattern regardless of the host tissue.14 It may become a useful indication for cases in which the superficial lamina propria layer requires recovery so as to correct free edge of VF irregularities (Figure 4).

This study demonstrated that HA Restylane® injected in rabbit VFs remains for up to three months, forming new connective tissue. Hallen (1999) states that new connective tissue increased with time of stay. This finding is in conflict with our results, where in the one week group 90% of rabbits had moderate fibrosis (falling to 10% on the third month); the percentage difference progressed to mild or absent fibrosis. These results show that fibrosis diminished with time, which is important to allow sliding of the lamina propria over the vocal muscle.

The receptor area affects the behavior of injected material. Rabbit VFs have a squamous stratified epithelium (four layers); below the epithelium there is a dense layer of collagen fibers, which some authors describe as the vocal ligament.^25^ There is loose connective tissue between the epithelium and the thyroarytenoid muscle, and its fundamental substance is rich in HA,^26^ similar to the superficial layer of the lamina propria in humans.^29^ Significant local mobility is undoubtedly a factor that may interfere with the success of HA application. This mobility may cause material to be expelled, with loss of the injected substance.^7^ Choice of the material should take into account physical property similarities with the application site. The material should also be able to mould itself, ameliorating the effects of mobility. It should meet local needs, namely resistance to stretching and to compression, and - in the specific case of VFs - the substance should attenuate impact during phonatory vibration (cushion effect). HA meets these VF-related physical property requirements.^16^

Mild inflammation, complete absence of foreign body reactions, absence of necrosis and granuloma formation, and negligible fibrogenesis observed with HA Restylane® during the study period, suggests that this is a potentially biocompatible and non-bioactive material. Complete integration of HA with VF tissues was not seen; thus, injected HA does not recompose the muco-ondulatory component, and has only a space filling function. Biocompatibility, ease of storage, an injectable form, and affordability suggests that HA Restylane® is a safe and valuable choice for the repair of anatomical alterations in VF stratification. In future this product may possibly be widely used in the larynx.

## CONCLUSION

1. Injection of HA Restylane® in rabbit VFs does not cause significant inflammation.
2. HA Restylane® remained in the rabbit VF for three months.

## ACKNOWLEDGEMENTS

We wish to thank the FCMSCSP biotherium team for the care given to the animals. We also wish to thank Fapesp for awarding credit to this study. We would also like to thank Ms Katya Karine Nery Carneiro for typing and formatting services.
